# Standardized, Individualized, or AI-Based Approach to Parenteral Nutrition in Neonatal Intensive Care Units: A Narrative Review

**DOI:** 10.7759/cureus.105328

**Published:** 2026-03-16

**Authors:** Weronika Walendziak, Karolina Domosud, Anna Malczyk, Damian Zienkiewicz, Gabriela Makulec, Kacper Ściebura, Magdalena Ostaszewska, Natalia Mordal, Wiktoria Wiśniewska, Milena Majchrzyk

**Affiliations:** 1 Medicine, National Medical Institute of the Ministry of the Interior and Administration, Warsaw, POL; 2 Medicine, Military Institute of Medicine - National Research Institute, Warsaw, POL; 3 Medicine, Międzyleski Specialist Hospital, Warsaw, POL; 4 Medicine, Praga Hospital of the Transfiguration of the Lord, Warsaw, POL; 5 Medicine, Mazovian Bródno Hospital, Warsaw, POL

**Keywords:** artificial intelligence in medicine, individualized parenteral nutrition, intensive care, neonatal care, neonatal intensive care, nutrition in critical care, parenteral nutrition (pn), preterm neonate, standardized parenteral nutrition, total parenteral nutrition (tpn)

## Abstract

Parenteral nutrition (PN) is fundamental in the management of premature infants hospitalized in neonatal intensive care units (NICUs). Traditionally, standardized PN (SPN) has been recommended for most newborns due to safety, faster initiation, and reduced risk of prescribing errors. However, individualized PN (IPN) remains necessary in cases of metabolic instability or prolonged PN. Recently, AI-based models, such as the TPN2.0 algorithm, have emerged as potential tools to enhance precision, safety, and clinical outcomes in PN by combining standardization and personalization. The aim of this review is to summarize and critically discuss current evidence regarding standardized, individualized, and AI-based approaches to PN in premature infants hospitalized in NICUs. Despite guidelines, practice varies, and the emerging evidence for AI needs critical synthesis. This narrative review is based on a focused, critical appraisal of the available literature. A nonsystematic search of PubMed/MEDLINE, Wiley Online Library, ScienceDirect, and Google Scholar was conducted to identify relevant articles. Additional sources included international clinical guidelines and consensus statements. The search focused on publications addressing standardized, individualized, and AI-supported PN in neonatal and pediatric intensive care settings. Selected studies were analyzed qualitatively with emphasis on clinical outcomes, nutritional adequacy, safety profiles, and practical implementation aspects. Due to the heterogeneity of study designs and outcomes, a formal systematic review methodology and meta-analysis were not performed. Across multiple studies, SPN has been found to provide adequate macronutrient and electrolyte intake for most NICU patients, enabling faster initiation of PN and reducing prescribing errors. SPN often resulted in improved early protein, glucose, calcium, and phosphate delivery compared with IPN, with fewer electrolyte disturbances and comparable or better growth outcomes. Evidence supporting IPN benefits has been inconsistent and limited primarily to metabolically unstable or complex cases. AI-based PN (TPN2.0) demonstrated promising results, with physicians rating its recommendations higher than standard prescriptions. In infants whose clinically prescribed PN differed most from AI recommendations, higher morbidity, including NEC, was observed compared with AI-guided formulations. The algorithm reduced formulation subjectivity, streamlined workflow, and enabled rapid, guideline-adherent PN prescription. Early evidence suggests a potential association with reduced rates of mortality, cholestasis, sepsis, and NEC with the use of AI-supported PN strategies. SPN remains the safest and most efficient first-line approach for most premature infants, ensuring rapid initiation and reducing prescription-related risks. IPN continues to be essential for selected high-risk patients with complex metabolic needs. Emerging AI-based systems such as TPN2.0 may bridge these approaches by delivering personalized yet SPN formulations, improving safety, efficiency, and potentially clinical outcomes. Further high-quality prospective trials are needed to validate these findings and support the integration of AI into routine NICU nutritional practice.

## Introduction and background

Parenteral nutrition (PN) is an intravenous method of providing essential nutrients required for growth and metabolism directly into the bloodstream. It serves as an alternative or complement to enteral feeding when the gastrointestinal tract is nonfunctional or cannot meet nutritional needs. PN solutions are formulated to match a patient’s specific requirements and are administered intravenously. Total PN (TPN) denotes a regimen in which PN provides the entirety of a patient’s caloric and nutrient requirements, typically in the absence of enteral nutrition or when enteral intake is limited to minimal amounts.

Individual PN requires frequent adjustments. The main advantage lies in its individualized preparation, designed to address the specific medical conditions of each patient and to provide optimal nutritional and biochemical outcomes [1,2]. However, this approach may encounter several limitations, including errors, stability issues, and increased risk of infections. Commercially produced PN bags are easily available as ward supplies in NICUs, which enables early PN initiation in premature infants. Furthermore, SPN solutions integrate nutritional knowledge of many experts and are included in the form of transparent guidelines, such as recommendations endorsed by the United Kingdom National Guideline Alliance [1].

Although standardized PN (SPN) remains the initial recommendation for most pediatric and neonatal cases, the personalization of nutritional management in intensive care units (ICUs) is crucial for advancing future critical care practice. In general, individually tailored PN solutions are required when the patient’s nutritional needs cannot be met with standard, pre-prepared formulations. Critically ill patients with severe metabolic imbalances, such as those with metabolic instability, may particularly benefit from this approach. It is also indicated for infants and children needing long-term support, for example, in cases of short bowel syndrome.

The primary challenge in personalizing care in the ICU and NICU is the difficulty of objectively assessing patients’ nutritional requirements and their physiological or clinical responses to nutritional therapy [2,3]. Modern technologies have the potential to enhance critical care nutrition by making it more scientifically rigorous, practically implementable, and objectively measurable. They facilitate more accurate estimation of nutritional needs and provide continuous monitoring of nutritional therapy through automated systems [4]. Technology can also enhance data analysis by leveraging electronic medical records, thereby advancing scientific understanding. AI has increasingly influenced modern healthcare in recent years. Implementing AI may become a breakthrough in improving patient care, with notable potential in the area of clinical nutrition.

In the following sections of this article, the outcomes of the abovementioned PN approaches were compared, considering indicators such as mortality, incidence of cholestasis, sepsis, and necrotizing enterocolitis, as well as the frequency of adverse effects in neonates requiring intensive care.

Recent studies evaluating the impact of personalized PN and SPN on neonatal outcomes in ICUs were also reviewed, and the clinical performance of the TPN2.0 AI algorithm was assessed. This algorithm uses clinical data to recommend the most appropriate nutritional regimen based on the individual clinical characteristics of each patient [5]. Through the integration of data-driven personalization and standardized practice, TPN2.0 effectively unifies both approaches.

## Review

Methods

This article is a narrative review of the literature focusing on different approaches to PN in neonatal ICUs (NICUs). A focused, nonsystematic literature search was conducted to identify publications relevant to standardized, individualized, and AI-supported PN in neonates.

**Figure 1:**
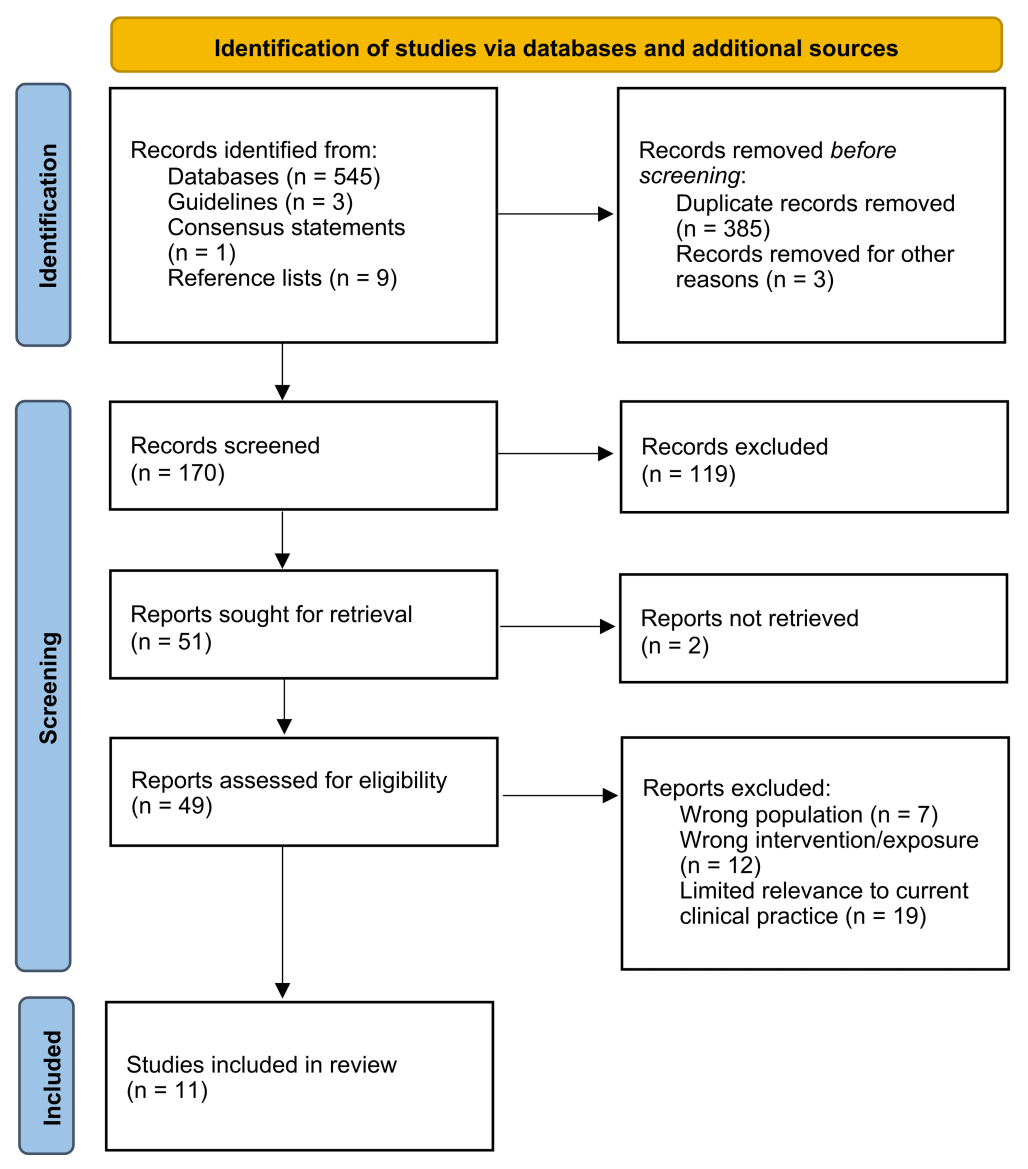
Literature search and selection process

The search was performed using the following electronic databases: PubMed/MEDLINE, ScienceDirect, Wiley Online Library, and Google Scholar. In addition, reference lists of key articles and international clinical guidelines and consensus documents were manually screened to identify additional relevant publications. Search terms included combinations of “parenteral nutrition”, “standardized”, “individualized”, “neonatal”, “preterm”, “artificial intelligence”, “machine learning”, “TPN”, and related Medical Subject Headings (MeSH) terms. No strict time limits were applied to capture both historical and recent developments in this field (Figure 1).

Publications were selected based on their relevance to the neonatal intensive care practice. Priority was given to randomized controlled trials (RCTs), observational cohort studies, retrospective analyses, clinical practice guidelines, and consensus statements. Articles focusing exclusively on adult populations or unrelated clinical contexts were excluded.

Data were extracted manually from selected publications and summarized descriptively. Extracted information included study design, patient population, type of PN strategy (standardized, individualized, or AI-supported), reported clinical outcomes (e.g., growth parameters, morbidity, mortality, metabolic stability), and organizational outcomes (e.g., time to initiation of nutrition, prescribing errors, and workflow efficiency).

To characterize AI-supported PN, the study describing the TPN2.0 algorithm was analyzed in detail [5]. In that study, the model was developed using a variational neural network combined with semi-supervised iterative clustering. The algorithm was trained on compressed electronic health record (EHR) features, daily laboratory values, demographic data, and key TPN parameters (including fluid goals and enteral intake) to generate recommended TPN formulations. The training dataset consisted of neonatal patients treated at Stanford Health Care (USA) between January 2011 and January 2022. External validation was performed in an independent cohort at the University of California, San Francisco, where the model was applied without retraining and analyzed independently.

Due to substantial heterogeneity across the included studies, a formal meta-analysis was not performed. The available evidence differed markedly with respect to study design (randomized trials, retrospective cohorts, and observational studies), patient populations (varying gestational age, birth weight, and clinical stability), and interventions (different standardized formulations, individualized compounding strategies, timing of initiation, and nutrient compositions). In addition, outcome reporting was inconsistent, encompassing diverse growth parameters, biochemical measures, nutrient intake calculations, and clinical endpoints assessed over noncomparable time frames. Because these methodological and clinical differences precluded meaningful pooling of effect estimates and risked generating misleading summary measures, the findings were synthesized narratively instead of quantitatively, with emphasis on consistency of results across studies, clinical relevance, and implications for practice. Because of the narrative character of this review, a formal systematic review methodology, a Preferred Reporting Items for Systematic reviews and Meta-Analyses (PRISMA)-guided workflow, and structured risk-of-bias assessment were not applied. As such, the review is not reported with sufficient methodological detail to allow for replication, which reflects an inherent limitation of narrative reviews.

Results

Guideline Recommendations

Neonatal parenteral nutrition guidelines by the National Institute for Health and Care Excellence (NICE) from 2020 provide a structured comparison between both approaches based on information obtained from several observational studies [6-8] and one RCT [9], which, despite its earlier publication date, was retained due to its methodological rigor and continued clinical relevance. These guidelines consider clinical evidence statements that have a significant impact on treatment outcomes. They comprise weight, weight loss, head circumference, sepsis, NEC, energy intake, administration of glucose, lipids, and proteins, nonprotein calorie delivery, duration of TPN, length of stay, and mortality [10]. These were identified as the factors most likely to be affected by differences between standardized and individualized PN (IPN). SPN was considered advantageous due to immediate availability, reduced prescribing errors, standardized quality control, and potential cost efficiency. The committee noted that positive outcomes for SPN would only be achievable if the formulation provided optimal nutrition. Other important outcomes included the duration of PN, prescribing errors, and length of hospital stay, although these were recognized as being influenced by multiple factors beyond the nutrition regimen itself. The available evidence did not include information on key aspects such as prescribing errors or the ease of PN administration. The committee also highlighted that SPN should be relatively concentrated to deliver adequate nutrients within neonatal fluid restrictions and that several standardized formulations are needed to meet varying clinical requirements. They considered SPN to be a safe, effective, and cost-efficient option, although some studies compared IPN with diluted SPN formulations, which may have underestimated the true effectiveness of well-designed SPN. The committee also recognized that IPN may still be appropriate in specific clinical situations, such as renal failure or complex gastrointestinal disorders. Finally, they found no biological or clinical rationale suggesting either SPN or IPN directly affects hospital stay duration but emphasized that standardized systems and protocols could improve consistency and quality of neonatal care across units. It should be noted that the evidence base was heterogeneous and of low quality, with substantial risk of bias and limited data for several clinically relevant outcomes, including neurodevelopment. As a result, the guidelines relied primarily on their clinical experience, current practice, and cost considerations to develop recommendations through informal consensus.

Current guidelines issued by the European Society for Paediatric Gastroenterology Hepatology and Nutrition (ESPGHAN), the European Society for Clinical Nutrition and Metabolism (ESPEN), the European Society for Paediatric Research (ESPR), and the Chinese Society for Parenteral and Enteral Nutrition (CSPEN) recommend the use of SPN solutions in the majority of pediatric and neonatal patients, including very low birth weight premature infants [11]. However, these recommendations are based on a low level of evidence, derived mainly from limited RCTs and retrospective observational studies, some of which are referenced in the Comparative Clinical Studies section [6,8,12-16]. IPN solutions should generally be reserved for cases in which nutritional requirements cannot be adequately met by standard PN formulations, for example, in critically ill and metabolically unstable patients with abnormal fluid or electrolyte losses, or in infants and children requiring prolonged PN support, such as those with short bowel syndrome. Also, electronic prescription, whether standardized or individualized, should be used in the process of ordering PN when possible [11].

In line with these findings, the Australasian Neonatal Parenteral Nutrition Consensus Group concluded that SPN offers clear benefits over IPN by ensuring adequate nutrition for most neonates in ICUs, maintaining stable biochemical outcomes, and potentially lowering costs and prescribing errors [6,8,17]. The Consensus Group concluded that SPN generally ensures adequate nutrition for the majority of neonates in NICUs without inducing significant biochemical alterations, while also offering potential advantages in terms of cost reduction and decreased risk of prescription errors.

In contrast to the previously described recommendations, the American Society for Parenteral and Enteral Nutrition (ASPEN) does not support the routine use of standard PN formulations in the care of preterm infants due to the absence of clinical trials directly evaluating this issue. This recommendation neither applies to nor discourages the use of commercially available ready-to-use PN solutions typically administered within the first 24 hours after birth (commonly referred to as “starter” or “backup” PN), which are valuable because of their immediate availability at any time [18]. According to ASPEN guidelines, SPN solutions cannot be formally recommended due to the current lack of clinical evidence. However, despite the absence of trial-based data, their relative ease of implementation may offer advantages in resource-limited settings.

Comparative Clinical Studies

Comparative clinical studies play a crucial role in evaluating different therapeutic strategies and assessing their effectiveness in clinical practice. By consistently comparing alternative approaches, these studies help identify optimal treatment models and support evidence-based decision-making.

In one study [12], no significant differences in weight gain were observed between premature infants receiving a standard solution and those given computer-generated individualized prescriptions. Another study [13] demonstrated that SPN was nutritionally adequate for most NICU patients, providing macronutrient and electrolyte intake comparable to IPN, while ensuring higher calcium and phosphate supply and fewer electrolyte imbalances.

A retrospective analysis [8] found no clinical or biochemical advantages of IPN between the second and seventh days of life. Infants receiving SPN had higher protein intake, smaller cumulative protein deficits (35% lower after the first week), greater calcium and phosphate provision, and improved bone mineralization. Similar findings were reported in a different study [6], which showed higher early amino acid and glucose intake and a more favorable calcium-to-phosphate ratio with SPN, without compromising biochemical stability.

A subsequent study [14] confirmed improved intake of protein, energy, glucose, and calcium, along with lower fluid volumes and fewer significant electrolyte disturbances in the SPN group. SPN has also been shown to effectively meet the nutritional requirements of preterm infants and children [15,16].

Observational studies suggest that implementing SPN is feasible and may provide clinical benefits in selected settings, although findings are not entirely consistent [6,8]. Reported advantages include higher energy and amino acid delivery, improved calcium and phosphate intake, reduced early postnatal weight loss, and lower costs. Evidence supporting superior growth outcomes with IPN remains limited and is confounded by temporal and management differences between cohorts [17].

The AI Model: TPN2.0

TPN2.0 represents a data-driven strategy for TPN, leveraging routinely gathered EHR information to optimize and standardize TPN practice [5]. Through this algorithm, 15 TPN formulations were identified, enabling a precision-medicine framework that may improve safety and reduce costs.

The model was developed to reduce subjectivity in formulation and enhance the efficiency of TPN compounding by using machine learning to generate a set of standardized TPN formulas and recommend the most appropriate option based on a patient’s clinical profile [5]. This data-driven approach minimizes the uncertainty surrounding optimal TPN composition, an area currently influenced by differing guidelines and individual provider experience. It simplifies the prescription workflow, decreasing the required human effort and reducing preparation time from several hours to only a few minutes. Importantly, this is accomplished while preserving the level of personalization needed to meet the diverse nutritional requirements of newborns. The introduction of standardized formulations also creates opportunities for mass manufacturing, eliminating the four- to 12-hour lead time and reducing errors associated with individualized compounding and shipping. Collectively, these advantages allow TPN2.0 to address both the limitations and accessibility challenges of current TPN processes.

Despite variability in TPN practices among providers and across health systems, shared underlying characteristics allowed TPN2.0 to achieve performance comparable to clinical experts at both sites. In a blinded study, physicians rated TPN2.0 more favorably than the current best practice [5]. Among patients with the greatest discrepancies between actual prescriptions and TPN2.0 recommendations, standard prescriptions were associated with higher morbidity, such as necrotizing enterocolitis, while TPN2.0-guided prescriptions were linked to a reduced risk. They also demonstrated that the transformer-based TPN2.0 system supports guideline-adherent, physician-in-the-loop recommendations, enabling effective collaboration between clinicians and AI [5].

Many prognostic models used in intensive care are highly accurate, but only a few have improved patient outcomes or changed clinical practice. In a setting with heavy workloads and large amounts of data, AI tools should provide clear, practical recommendations rather than predictions alone. TPN is an area where such support is especially needed and possible to implement. This is particularly true in newborns, whose complex nutritional needs and limited guidelines often require clinicians to rely on personal experience. In addition, prescribing and preparing TPN is time-consuming, involves multiple professionals, and carries a high risk of errors.

The solutions offered by TPN2.0, such as its data-driven structure, standardization, and objective recommendations, directly address these challenges. The system combines personalization with standardized protocols, using evidence-based guidance that has been associated with improved clinical outcomes, including reduced morbidity. The set of 15 standardized formulas enhances safety and lowers production costs, while the AI component responds to the dynamic needs of infants during their NICU stay [5].

Overall, TPN2.0 demonstrates how AI can move beyond predictive tasks to support clinicians in making key therapeutic decisions during the most vulnerable period of a newborn’s life. This approach optimizes and standardizes TPN management, enhances safety, and increases accessibility, particularly in resource-limited settings.

Discussion

Synthesis of Main Findings (SPN/IPN)

Across the reviewed studies, no consistent superiority of individualized over SPN has been demonstrated with respect to growth. Weight gain is generally comparable between approaches, although SPN appears to reduce the early cumulative protein deficit in neonatal life. Regarding nutrient delivery, SPN consistently provides higher protein intake, improved calcium and phosphate provision, and more adequate early nutritional support. In terms of safety and biochemical outcomes, SPN is associated with fewer electrolyte disturbances, does not compromise biochemical stability, and, according to guideline assessments, carries a lower risk of prescribing errors. From a practical perspective, SPN enables faster initiation of PN due to ready-to-use formulations and may reduce costs, as reported in consensus statements. By contrast, IPN remains particularly useful in complex metabolic situations requiring highly individualized management. Overall, findings from both guidelines and comparative clinical studies support SPN as the default strategy for neonatal PN, whereas IPN should be reserved for selected clinical circumstances. Nevertheless, the overall quality of evidence is low, RCTs are scarce, and no clear superiority of either approach in terms of growth outcomes has been definitively established (Table 1, Table 2).

**Table 1:** Guideline and consensus recommendations (SPN vs IPN) IPN, individualized parenteral nutrition; RCT, randomized controlled trial; SPN, standardized parenteral nutrition

Study	Guideline or group	Evidence base	SPN vs IPN: position and rationale	Important notes
National Guideline Alliance (UK) (2020) [10]	NICE	Observational studies + one RCT, overall low-quality evidence	Strongly favors SPN due to immediate availability, fewer prescribing errors, faster initiation of nutrition, improved compliance, and cost efficiency	No neurodevelopment data, recommendations partly based on expert consensus
Riskin et al. (2018) [11]	ESPGHAN/ESPEN/ESPR/CSPEN	Limited RCTs + retrospective studies	SPN recommended for most neonates, providing adequate nutrition for the majority of patients	IPN reserved for unstable or complex cases
Bolisetty et al. (2014) [17]	Australasian Neonatal Parenteral Nutrition Consensus Group	Observational evidence	SPN more beneficial than IPN, offering adequate nutrition, a stable biochemical profile, fewer prescribing errors, and lower costs	Benefits not universal
Robinson et al. (2023) [18]	ASPEN	Insufficient direct clinical trials	Does not formally recommend routine SPN due to a lack of high-quality trials comparing growth outcomes	Starter SPN acceptable due to immediate availability

**Table 2:** Comparative clinical studies (SPN vs IPN) IPN, individualized parenteral nutrition; NICU, neonatal intensive care unit; SPN, standardized parenteral nutrition

Reference	Study design	Population	Main outcomes measured	Main findings
Lenclen et al. (2006) [6]	Observational	Preterm infants	Early nutrient intake, Ca:P ratio	SPN: higher amino acid and glucose intake, improved Ca:P ratio, and stable biochemical parameters
Yeung et al. (2003) [8]	Retrospective cohort (days 2-7)	Preterm infants	Nutrient intake, biochemical outcomes	No benefit of IPN, with SPN providing higher protein intake and a 35% lower protein deficit
Cade et al. (1997) [12]	Comparative clinical study	Premature infants	Weight gain	No significant difference between SPN and computer-generated IPN
Krohn et al. (2005) [13]	Observational NICU study	NICU neonates	Nutrient adequacy, electrolytes	Similar macronutrient intake in both IPN and SPN, with SPN providing higher Ca/P intake and fewer electrolyte imbalances
Skouroliakou et al. (2009) [14]	Observational	Premature infants	Intake and electrolyte disturbances	SPN: higher protein, energy, glucose, and calcium intake, with fewer electrolyte imbalances
Caba Porras et al. (2010) [15]; Rigo and Senterre (2013) [16]	Clinical feasibility studies	Preterm infants and children	Nutritional goal achievement	Nutritional requirements effectively met with SPN

Practical Implementation and Broader AI Applications

Despite the continued popularity of all-in-one formulations, recent years have seen increasing investment by hospitals in in-house PN laboratories. This trend is partly driven by the development of machine learning algorithms applied in NICU care [2]. Contemporary TPN emphasizes individualized therapy that accounts for age, body weight, previous diseases, metabolic requirements, and comorbidities. The objective of targeted PN is to prevent both overfeeding and underfeeding, as each is associated with increased morbidity [19]. In critically ill patients, dynamic modification of nutritional therapy based on current clinical status and laboratory findings has become standard practice [20].

Neonatal supplementation presents additional complexity because it requires continuous infusion of all components over prolonged periods to meet metabolic demands. In many healthcare settings, vitamins and trace elements are added to the admixture immediately before administration to optimize stability and efficacy [21]. Preterm infants in particular require highly specialized TPN that supports rapid growth while minimizing complications such as hyperglycemia and osteopenia of prematurity. Under these conditions, SPN is frequently insufficient. The recently developed TPN 2.0 system, which utilizes data from EHRs, further enhances formulation accuracy and adaptability, improving the capacity to meet the nutritional requirements of neonatal patients.

Technological advancements, including computerized provider order entry (CPOE), EHRs, and clinical decision support (CDS) systems, have contributed to a reduction in the risk of errors during the prescription, preparation, and administration of PN by guiding prescribing processes, performing automated safety checks, and improving the accuracy and safety of PN ordering, compounding, and delivery. However, limited adoption and suboptimal optimization of electronic PN systems may prevent full realization of their safety potential, allowing errors such as transcription mistakes, dosing inaccuracies, and component incompatibilities to persist, with potentially serious consequences for patient outcomes [22]. Nevertheless, it is generally accepted that the implementation of these technologies has markedly enhanced the quality of PN, particularly in neonatal and pediatric patients, where precise adjustment of nutritional therapy is especially critical.

There are several ways in which PN automation can enhance the workflow of healthcare professionals and facilities. These systems ensure the precise preparation of nutritional mixtures by accurately dosing electrolytes, amino acids, lipids, glucose, vitamins, and micronutrients, essential for maintaining high safety standards and consistency. Additionally, they automate the compounding process, thereby minimizing the risk of human error and enabling rapid production of formulations, especially in ICU settings. Integration with hospital pharmacy management systems and electronic medical records allows for efficient monitoring of nutrient delivery and order history. Altogether, these functions reduce the workload of medical staff, reduce preparation time, and facilitate swift responses to changing clinical conditions. However, in most hospitals, prescriptions are still reviewed by prescription-reviewing pharmacists [23].

Numerous studies have demonstrated the effectiveness of automated TPN systems. In one analysis, the accuracy of the calculation formula and the performance of the electronic prescription reviewing system were evaluated [23]. The introduction of these two systems resulted in improvement in the work efficiency of prescription checking, patient medication safety was better secured, and the defective rate in PN prescriptions was remarkably decreased. The use of a commercially available CPOE system to manage PN ordering was analyzed in an academic medical center encompassing three hospitals with 1,000 licensed beds [22]. The findings suggest that carefully planned electronic PN ordering systems and accompanying CDS can enhance the accuracy, safety, and efficiency of this complex process. However, achieving these benefits requires substantial initial and continuous technical support. The authors concluded that ongoing maintenance of these systems is essential to account for new medications, updated products, fluctuating drug shortages, and emerging evidence on compatibility and safety. At a large academic pediatric hospital, standardized TPN formulations for neonatal and pediatric patients ranging from birth to 21 years of age were introduced [24]. The project implemented four key interventions: transitioning from paper to electronic ordering, providing standardized TPN solutions, creating standardized laboratory draw schedules, and having predetermined acceptable laboratory parameters. These combined measures led to a significant reduction in ordering errors and processing time, while also improving resource efficiency by decreasing the number of required blood draws.

Beyond the algorithms used to formulate PN solutions in the ICU and NICU settings, machine-learning-based tools have also demonstrated utility in other aspects of critical care management, including predicting enteral feeding intolerance (EFI) [25], assessing the prognostic value of EFI markers [26], forecasting gastrointestinal symptoms in ICU patients [27], detecting patients at risk of refeeding hypophosphatemia [28], and determining the optimal timing for initiating enteral nutrition [29].

Future Directions 

The use of modern technologies has made nutrition support in the ICU significantly simpler, more objective, and faster to implement. Automated systems assist in estimating a patient’s energy requirements, predicting protein and calorie utilization, and providing real-time monitoring of nutrient delivery. Another important step in applying new technologies to clinical nutrition is the analysis of data through electronic medical records, which supports the expansion of scientific knowledge and facilitates research. In recent years, healthcare has undergone remarkable progress driven by the integration of AI into many areas of medical practice. AI has emerged as a promising tool with substantial potential to improve patient care, including in medical nutrition support. The growing role of AI in this field may provide a novel therapeutic avenue for patients with special nutritional needs by enabling more precise and better-balanced dietary interventions that can enhance quality of life and clinical outcomes. This is especially crucial for ICU and NICU patients, whose nutritional status directly influences both the pace and the likelihood of recovery [30].

On the other hand, the implementation of machine learning in clinical nutrition is heavily dependent on large, well-curated datasets. Its effective application requires systematic data collection, integration, storage, and ongoing maintenance, which can impose substantial financial and logistical burdens on healthcare institutions, including costs related to hardware and cloud computing. The heterogeneity of software platforms and programming languages further complicates standardization efforts. Moreover, successful deployment necessitates close collaboration between specialized data scientists and experienced clinicians to ensure that predictive models are both technically robust and clinically meaningful. Further studies are needed to assess the actual role and impact of AI in clinical nutrition and to explore future directions for its safe, effective, and evidence-based integration into ICU and NICU care.

## Conclusions

This narrative review examines three approaches to PN in NICUs: SPN, IPN, and AI-guided strategies. Although NICUs have increasingly explored individualized nutritional management, current evidence and major guidelines (NICE, ESPGHAN/ESPEN/ESPR/CSPEN, and the Australasian consensus) generally support SPN as the first-line strategy for most premature and critically ill neonates. The findings indicate that SPN enables earlier initiation of nutritional support, improves consistency of nutrient delivery, reduces prescribing and compounding errors, and is more cost-effective. Compared with IPN, SPN is associated with comparable or superior delivery of protein, energy, calcium, and phosphate, fewer electrolyte imbalances, and similar or improved growth outcomes. IPN remains valuable in selected clinical scenarios, such as severe metabolic instability, renal failure, or long-term PN requirements (e.g., short bowel syndrome). However, the overall quality of the available evidence remains low, with many studies at risk of bias. The increasing use of automation and electronic systems (CPOE, EHR, and CDS tools) is associated with a reduction in PN-related errors, improved clinical workflow, and enhanced patient safety. Particular attention has been given to the emerging AI-based model TPN2.0, which uses machine learning to recommend standardized yet personalized PN formulations. Available studies suggest that AI-guided PN may be associated with lower morbidity, including reduced rates of necrotizing enterocolitis, cholestasis, and sepsis, and is frequently rated more favorably by clinicians than traditional prescribing practices. Overall, SPN should be considered the standard approach for most NICU patients, with IPN reserved for complex cases, while AI-assisted systems such as TPN2.0 represent a promising future direction by integrating safety, standardization, and personalization to improve neonatal outcomes.
